# Aqueous extract from the *Withania somnifera* leaves as a potential anti-neuroinflammatory agent: a mechanistic study

**DOI:** 10.1186/s12974-016-0650-3

**Published:** 2016-08-22

**Authors:** Muskan Gupta, Gurcharan Kaur

**Affiliations:** Department of Biotechnology, Guru Nanak Dev University, Amritsar, Punjab 143005 India

**Keywords:** Ashwagandha, Microglia, Neuroinflammation, Apoptosis, Pro-inflammatory mediators, Inflammatory cytokines

## Abstract

**Background:**

Microglial-mediated neuroinflammation is a key factor underlying the pathogenesis of various neurodegenerative diseases and also an important target for the development of the neuroinflammation-targeted therapeutics. Conventionally, the nonsteroidal anti-inflammatory drugs (NSAIDs) are prescribed, but they are associated with long-term potential risks. Natural products are the cornerstone of modern therapeutics, and Ashwagandha is one such plant which is well known for its immunomodulatory properties in Ayurveda.

**Methods:**

The current study was aimed to investigate the anti-neuroinflammatory potential of Ashwagandha (*Withania somnifera*) leaf water extract (ASH-WEX) and one of its active chloroform fraction (fraction IV (FIV)) using β-amyloid and lipopolysaccharide (LPS)-stimulated primary microglial cells and BV-2 microglial cell line. Iba-1 and α-tubulin immunocytochemistry was done to study the LPS- and β-amyloid-induced morphological changes in microglial cells. Inflammatory molecules (NFkB, AP1), oxidative stress proteins (HSP 70, mortalin), apoptotic markers (Bcl-xl, PARP), cell cycle regulatory proteins (PCNA, Cyclin D1), and MHC II expression were analyzed by Western blotting. Mitotracker and CellRox Staining, Sandwich ELISA, and Gelatin Zymography were done to investigate ROS, pro-inflammatory cytokines, and matrix metalloproteinase production, respectively. Ashwagandha effect on microglial proliferation, migration, and its apoptosis-inducing potential was studied by cell cycle analysis, migration assay, and Annexin-V FITC assay, respectively.

**Results:**

ASH-WEX and FIV pretreatment was seen to suppress the proliferation of activated microglia by causing cell cycle arrest at Go/G1 and G2/M phase along with decrease in cell cycle regulatory protein expression such as PCNA and Cyclin D1. Inhibition of microglial activation was revealed by their morphology and downregulated expression of microglial activation markers like MHC II and Iba-1. Both the extracts attenuated the TNF-α, IL-1β, IL-6, RNS, and ROS production via downregulating the expression of inflammatory proteins like NFkB and AP1. ASH-WEX and FIV also restricted the migration of activated microglia by downregulating metalloproteinase expression. Controlled proliferation rate was also accompanied by apoptosis of activated microglia. ASH-WEX and FIV were screened and found to possess Withaferin A and Withanone as active phytochemicals.

**Conclusions:**

The current data suggests that ASH-WEX and FIV inhibit microglial activation and migration and may prove to be a potential therapeutic candidate for the suppression of neuroinflammation in the treatment of neurodegenerative diseases.

## Background

Neuroinflammation accompanies all neurological afflictions from traumatic brain injuries and nervous system infections to chronic neurodegenerative diseases. Microglia, resident immune cells in the brain, are the key contributors of this neuroinflammation resulting in neurodegeneration. The microglial-mediated neuroinflammation has been the characteristic diagnostic feature of various neurodegenerative diseases, including Alzheimer’s disease (AD), Parkinson’s disease (PD), trauma, multiple sclerosis (MS), and cerebral ischemia [1]. Under normal physiological conditions, these special brain macrophages serve the role of immune surveillance displaying M2 phenotype by secreting various neurotrophic and anti-inflammatory factors creating protective microenvironment for the neurons. However, in response to infection or injury, they readily become activated and converted in the form of M1 phenotype displaying a variety of surface receptors, including the MHCs and complement receptors [2]. They also undergo dramatic morphological changes from resting ramified cells to activated amoeboid microglia [3]. Chronic activation of microglia results in neuroinflammation by secreting various neurotoxic and pro-inflammatory mediators causing demyelination and neuronal death [4]. These mediators include PGE_2_, monocyte chemoattractant protein-1 (MCP-1), inflammatory cytokines such as interleukin-1 β (IL-1β), IL-6, and tumor necrosis factor-α (TNF-α) and free radicals such as nitric oxide (NO) and superoxide, fatty acid metabolites such as eicosanoids, and quinolinic acid [5].

Conventionally, various nonsteroidal anti-inflammatory drugs (NSAIDs) like rofecoxib, COX 2 inhibitors, aspirin, mefenamic acid, indomethacin, and ketoprofen are used to treat neurodegenerative diseases [6, 7]. However, treatment with NSAIDs is generally accompanied by various side effects like gastrointestinal problems, dizziness, headache, and dyspepsia as their major drawback. Recently, many medicinal herbs and their bioactive components like epigallocatechin-3-gallate (EGCG) from *Camellia sinensis*, curcumin from *Cucurma longa*, and resveratrol from grapes have been reported to possess anti-inflammatory activity and are more safe, effective, and easy to use than the NSAIDs with negligible potential risks [8–11].

*Withania somnifera* is the most popular medicinal plant in Ayurveda for its nerve tonic and memory enhancing properties. It is also well acknowledged for its longevity and vitality increasing properties [12]. Ashwagandha has been reported to possess a number of therapeutic properties including anti-inflammatory, sedative, hypnotic, narcotic, and general tonic [13]. The root extract of *Withania* has been found to be effective in protecting against hydrogen peroxide- and β-amyloid (1–42)-induced cytotoxicity in NGF-differentiated PC12 cells [14]. Withania contains active ingredients like steroidal alkaloids and lactones known as “withanolides.” Among the bioactive components of *Withania*, Withaferin A (Wit A) and withanolide D are well characterized which contribute to the most of the pharmaceutical properties of this plant [15]. Wit A has been shown to exhibit anti-inflammatory acitivity via NF-kB activation inhibition by blocking IkB phosphorylation and inhibiting IkB kinase activation [16, 17]. It also inhibited lipopolysaccharide (LPS)-induced PGE_2_ production and COX-2 expression in BV-2 cells and primary microglia, and these effects are mediated, at least in part, by reduced phosphorylation and nuclear translocation of STAT1 [18].

The anticancerous activity of Ashwagandha has already been well documented by our lab using both in vitro and in vivo model systems [19–21]. Further, it has also been shown to have neuroprotective potential against glutamate cytotoxicity [22].

In view of the previous reports, the present study was planned to further elucidate anti-neuroinflammatory potential of Ashwagandha leaf water extract (ASH-WEX) and one of its active chloroform fraction (fraction IV (FIV)) in LPS-induced primary microglial cells and BV-2 murine microglial cell line as an in vitro model system. LPS-induced activation of the microglial cells is a useful model for the study of neuroinflammation and nerve cell injury as they release various pro-inflammatory and neurotoxic factors [23]. We have also done preliminary study using β-amyloid-activated BV-2 and primary microglial cells as the inflammatory model to confirm the anti-neuroinflammatory potential of ASH-WEX and FIV in alternative condition. β-amyloid both recruits and activates the microglia, thereby initiating vicious cycle of the inflammation between β-amyloid accumulation, activated microglia and microglial inflammatory mediators, which enhance β-amyloid deposition and neuroinflammation thus considered as the unifying factor in the development of AD [24]. Further, we examined the signaling cascades associated with ASH-WEX and FIV on the proliferation and migration of LPS-induced microglial cells and their phenotypic changes which have been found to be responsible for neuronal damage in various neurological disorders. We further studied the effect of ASH-WEX and FIV on various inflammatory pathways like nuclear factor-kB (NFkB), activator protein 1 (AP1), and pAkt^SER-473^ which are known to mediate the production of various pro-inflammatory mediators like reactive nitrogen species (RNS), reactive oxygen species (ROS), inflammatory cytokines, and matrix metalloproteinases (MMPs) as a result of microglial cell activation.

## Methods

### Ashwagandha leaf extract

The ASH-WEX was prepared by suspending 10 g of dry leaf powder of Ashwagandha in 100 ml of water and stirring it overnight at 45 °C in shaker incubator. The suspension was then filtered under sterile conditions to obtain the aqueous leaf extract. The filtrate obtained was treated as 100 % aqueous extract and diluted in Dulbecco’s modified Eagle’s medium (DMEM) with 10 % fetal bovine serum (FBS) according to experimental requirement.

One of its fractions was prepared by solvent extraction in the following order: hexane → chloroform → ethylacetate → butanol. Then, the active chloroform fraction was further fractionated by thin-layer chromatography using hexane: ethylacetate as the mobile phase and four fractions were obtained. Out of these, one fraction was active in suppressing LPS-induced microglial activation and was labeled as the FIV. It was then dried and dissolved in DMSO at the concentration of the 50 mg/ml and diluted in DMEM according to the experimental requirement. ASH-WEX and FIV were also analyzed for the presence of the possible bioactive components Wit A and Withanone by reverse-phase chromatography. For HPLC, the samples were prepared by dissolving dried ASH-WEX and FIV in methanol at 10 and 2 mg/ml concentration, respectively. Purified Wit A and Withanone dissolved in methanol were used as the standards to determine their concentration in ASH-WEX and FIV.

### Primary microglial cultures

Primary microglia-enriched cultures were obtained from primary mixed glial cultures from 1-day-old Wistar rat pups. Animal care and procedures were followed in accordance with the guidelines of the Animal Ethical Committee, Guru Nanak Dev University, Amritsar, India. To obtain mixed glial cultures, the brain was dissected; their meninges were carefully removed and digested with 0.05 % trypsin-EDTA solution for 10 min at 37 °C. Trypsinization was stopped by adding an equal volume of DMEM culture medium supplemented with 10 % FBS. Cells were pelleted (5 min, 200 g), resuspended in culture medium, and brought to a single-cell suspension by repeated pipetting. Cells were seeded and cultured at 37 °C in a 5 % CO_2_ humidified atmosphere. Medium was replaced after every 3 days. Microglial cultures were prepared by the mild trypsinization method [25]. Briefly, after 18–20 days, in vitro (DIV) mixed glial cultures were treated for 30 min with 0.25 % trypsin containing 1 mM EDTA diluted with DMEM containing 1 mM Ca^2^+ in 1:3 ratio. This resulted in the detachment of an intact layer of cells containing virtually all the astrocytes, leaving a population of firmly attached cells identified as 98 % microglia. Isolated microglial cells were seeded in multiwell plates by trypsinization with 0.25 % trypsin-EDTA for 10 min and treated after 24 h of seeding.

### BV-2 cell culture and treatments

BV-2 cell line was obtained from National Brain Research Centre (NBRC), Manesar, Haryana, India. BV-2 cells are murine-cultured microglial cells immortalized after transfection with a v-raf/v-myc recombinant retrovirus. The cells were maintained in DMEM medium with 10 % FBS (Biological Industries) and 1× PSN mix (Invitrogen, Carlsbad, CA, USA) at 37 °C in 5 % CO_2_ and humid environment.

Both primary and BV-2 microglial cultures were pretreated with ASH-WEX and FIV for 2 h before adding 100 ng/ml LPS to cultures (LPS (rough strains) from *Escherichia coli* F583 (Rd mutant) and 5 nM β-amyloid (Sigma-Aldrich, Saint Louis, Missouri, 63103 USA). Treatment was done for 36 h before harvesting for different assays.

### Cytotoxicity and cell viability assays

ASH-WEX and one of its active fraction (FIV) were tested for cell viability using the 3-(4,5-dimethylthiazol-2-yl)-2,5-diphenyltetrazolium bromide (MTT) test. The cell viability was quantified by the conversion of yellow MTT by mitochondrial dehydrogenases of living cells to purple MTT formazan at 595-nm wavelength [26].

### Cell morphology studies

Morphological changes in both primary and BV-2 microglial cells treated with different concentrations of ASH-WEX and FIV with or without activation with LPS and β-amyloid were studied by phase contrast microscopy. These experiments were performed in 24-well plates containing poly-l-lysine-coated coverslips (20,000 cells/ml). The phase contrast images of culture were taken using Phase Contrast Inverted Microscope (Nikon TE2000). Nuclear morphology was studied by staining the nucleus with an AT-rich region-specific fluorescent stain, i.e., 4′,6-diamidino-2-phenylindole (DAPI).

### Immunostaining

Both primary and BV-2 microglial cells, control and treated, were washed with 1× phosphate-buffered saline (PBS) thrice and were fixed with acetone to methanol followed by permeabilization with 0.3 % Triton X-100 in PBS (PBST). Cells were then incubated with anti-α-tubulin (1:500) or anti-NF-kB (1:300) or anti-AP1 (1:300) or anti-HSP-70 (1:300) (from Sigma-Aldrich) diluted in 2 % BSA, for 24 h at 4 °C in humid chamber. After two to three washings with 0.1 % PBST, the cells were incubated with the secondary antibody anti-mouse IgG 488 and anti-rabbit IgG 488 (prepared in 2 % BSA (1:500)) for 2 h at room temperature. Cells were incubated with (DAPI, 1:5000 in 1× PBS) for 10 min for nuclear staining and then mounted with anti-fading reagent (Fluoromount, Sigma). For CellRox and Mitotracker staining, after treatment period, the dye was added in culture according to the manufacturer’s instructions; cells were then fixed and mounted. Images were captured using Nikon AIR Confocal Laser Microscope and analyzed using NIS elements AR analysis software version 4.11.00. Experiment was performed in triplicate.

### Protein assay and Western blotting

For total protein extraction, BV-2 microglial cells were grown and treated in 100-mm petri dishes followed by harvesting with PBS-EDTA (1 mM). Nuclear and cytoplasmic protein fractionation was performed using a CelLytic NuCLEAR extraction kit from Sigma-Aldrich. Briefly, the cell pellet obtained was allowed to swell with hypotonic buffer (100 mM HEPES, pH 7.9, 15 mM MgCl_2_, and 100 mM KCl). To the swollen cells in lysis buffer, 10 % IGEPAL CA-630 solution was added to a final concentration of 0.6 % and vortexed vigorously for 10 s. The cells are then disrupted, the cytoplasmic fraction was removed, and the nuclear proteins were released from the nuclei by a high-salt buffer (20 mM HEPES, pH 7.9, with 1.5 mM MgCl_2_, 0.42 M NaCl, 0.2 mM EDTA, and 25 % (*v*/*v*) glycerol). For total protein extraction, the cell pellet was homogenized in RIPA buffer (50 mM Tris (pH 7.5), 150 mM NaCl, 0.5 % sodium deoxycholate, 0.1 % sodium dodecyl sulfate (SDS), 1.0 % NP-40). Protein concentration was determined by the Bradford method. Protein lysate (30–50 μg) was resolved by SDS-polyacrylamide gel electrophoresis (SDS-PAGE), followed by transfer onto a PVDF membrane (Hybond-P) using the semi-dry Novablot system (Amersham Pharmacia). Further, membranes were probed with anti-mortalin (1:1000), anti-HSP70 (1:2500), anti-NFkB (1:2500), anti-OX-42 (1:1000), anti-OX-6 (1:1000), anti-PARP (1:1500), anti-Bcl-xl (1:1000), anti-AP1 (1:2500), anti-Akt (1:2500), and anti-Cyclin D1 (1:2000) (Sigma-Aldrich) and anti-proliferating cell nuclear antigen (PCNA) (1:2000) (Chemicon International, Temecula, CA, USA), for overnight at 4 °C. This was followed by washing with 0.1 % TBST and incubation with HRP-labeled secondary antibodies for 2 h at room temperature. Immunoreactive bands were detected by ECL Plus Western blot detection system (Amersham Biosciences) using LAS 4000 (GE Biosciences). α-Tubulin has been used as an endogenous control for normalizing the expression of the protein of interest. α-Tubulin and histone 3 were used as internal control for cytoplasmic and nuclear expression of proteins of interest, respectively. The change in expression of gene of interests was taken on the average of IDV values obtained from at least three independent experiments.

### Cell cycle analysis

BV-2 microglial cells were seeded at the cell density 2.5 × 10^5^ per ml in 100-mm-diameter petri plates and then treated according to the regime for 36 h. Cells from four petri dishes were pooled together. After 36 h of treatment, cells were trypsinized and then centrifuged at 1000 rpm. The cell pellet was resuspended in 1 ml of ice-cold PBS and then fixed with ice-cold 70 % ethanol for 2 h at 4 °C. Cells were then centrifuged and resuspended in 1 ml of PBS and incubated for 15 min and again centrifuged and resuspended in propidium iodide (PI) staining solution (100 mM Tris pH 7.4, 150 mM CaCl_2_, 0.5 mM MgCl_2_, 0.1 % NP-40, and 3 μM PI) and scanned with BD Accuri C6 Flow cytometer (BD Biosciences). DNA content histograms and cell cycle phase distribution were analyzed from at least 50,000 single events by excluding cell aggregates based on scatter plots of fluorescence pulse area versus fluorescence pulse width using FCS Express 4 flow research edition software (De Novo software).

### Annexin V-FITC (apoptosis) assay

To further explore whether ASH-WEX and FIV caused the apoptosis of the activated BV-2 microglia, cell suspension was harvested from cells grown and treated in 100 mm petris. Cells from the four petri dishes were pooled together. Cells were stained with Annexin V conjugated with FITC and PI using Annexin V-FITC Apoptosis Detection Kit (Miltenyi Biotech, San Diego, CA, USA). Briefly, 1 ml of 1× binding buffer was added to cells, and pellet was obtained after centrifugation at 5000 rpm. One hundred microliters of 1× binding buffer and 10 μl Annexin V-FITC was added to it and incubated for 15 min in the dark. Pellet was obtained by centrifugation at 5000 rpm after adding 1 ml 1× binding buffer and resuspended in 500 μl of 1× binding buffer. Five microliters of propidine iodide solution was added immediately prior to analysis by BD C6 Accuri flow cytometer.

### Wound scratch assay

In order to test anti-migration potential of ASH-WEX and FIV, BV-2 microglial cells were grown to confluent monolayer and then wounded by scratching the surface with a pipette tip. Cells were firstly pretreated with the ASH-WEX and FIV followed by activation with LPS. The initial wounding and the migration of cells in the scratched area were photographically monitored for 12–24 h after the treatment. Images were analyzed by Image Pro Plus software version 4.5.1 from the media cybernetics for the migration of cells in the scratched area by calculating the distance covered by the invading cells after incubation. Experiment was repeated for at least three times in triplicate.

### Gelatin zymogram study

In order to study the effect of ASH-WEX and FIV on matrix metalloproteinase (MMP 2, MMP 9)-conditioned media from both primary and BV-2 microglial cell cultures after treatment were separated on a 10 % SDS-PAGE containing 0.1 % gelatin. After electrophoresis, gels were washed with renaturation buffer (Invitrogen) for 1 h to remove SDS. The gel was then incubated in zymogram developing buffer (Invitrogen) at 37 °C for 48 h followed by staining with Coomassie brilliant blue and destaining in buffer containing 50 % methanol and 10 % acetic acid (*v*/*v*). The location of gelatinolytic activity was detected as clear white bands.

### Nitrite determination

Both primary and BV-2 microglial cells were seeded and treated as per the regimen. Nitrite determination was done using the Griess reagent nitrite determination kit (Molecular Probes, Invitrogen) as per the manufacturer’s protocol. Briefly, the culture conditioned media was collected and mixed with the Griess reagent, and the readings were obtained at the 548-nm wavelength. The nitrite concentrations were calculated using the sodium nitrite standard curve.

### Pro-inflammatory cytokine assay

Cell culture conditioned media was collected from the different treatment conditions and analyzed for the presence of the pro-inflammatory cytokines (IL-1β, IL-6, TNF-α) using the pro-inflammatory cytokine sandwich ELISA-based kits (from Sigma-Aldrich).

### Statistical analysis

Values are expressed as mean ± standard error of the mean (SEM) obtained from at least three independent experiments. The Sigma Stat for Windows (version 3.5) was used to analyze the results by one-way ANOVA (Holm-Sidak post hoc method), in order to determine the significance of the mean values. Values of *p* < 0.05 were considered as statistically significant.

## Results

### Standardization of ASH-WEX and FIV effective concentration

**Fig. 1:**
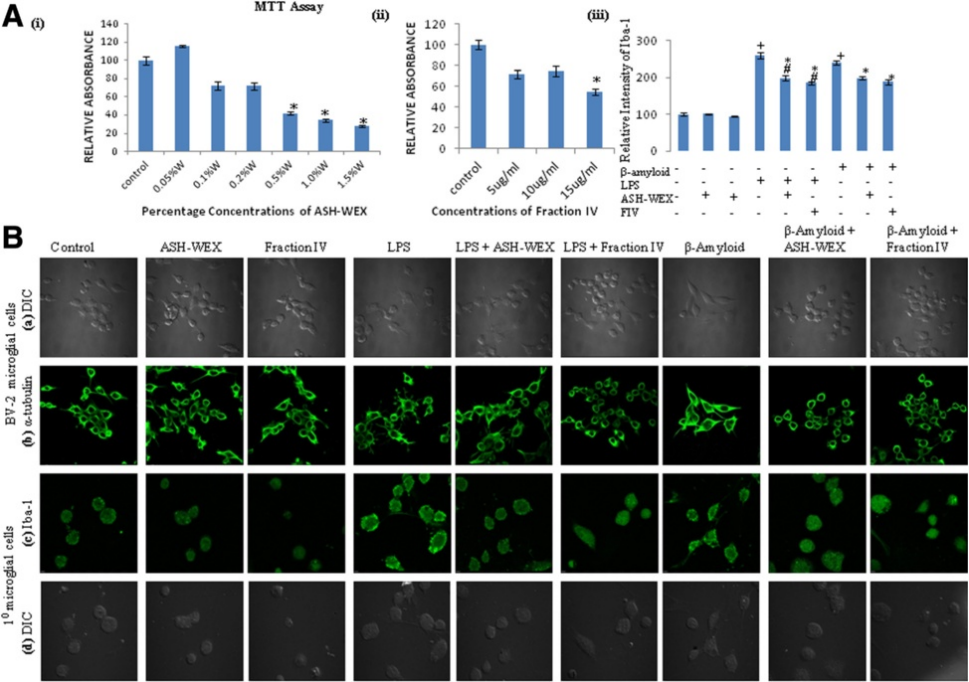
ASH-WEX and FIV pretreatment inhibits both β-amyloid and LPS-induced morphological changes in primary and BV-2 microglia. **A** Histograms represent the cytotoxicity assay of the BV-2 microglia treated with the (**i**) ASH-WEX and (**ii**) FIV after 48 h of the treatment. ASH-WEX and FIV inhibit the change in the microglial morphology from ramified to amoeboid due to LPS and β-amyloid treatment. **iii** Histogram represents the relative optical intensity of Iba-1 in primary microglial cells among the different treated groups. **B** (**a**) Represents the differential interference contrast (*DIC*) images of BV-2 microglial cells pretreated with ASH-WEX and FIV with or without treatment with LPS and β-amyloid, (**b**) Confocal images of α-tubulin immunostaining of the BV-2 microglia pretreated with ASH-WEX and FIV with or without activation with LPS and β-amyloid showing changes in the morphology, (**c**) Confocal images of Iba-1 immunostaining of primary microglial cells pretreated with ASH-WEX and FIV with or without treatment with LPS and β-amyloid, a specific microglial marker allowing distinction between activated and resting microglia in primary microglial cells, and (**d**) DIC images of primary microglial cells pretreated with ASH-WEX and FIV with or without treatment with LPS and β-amyloid (*scale bar* 50 μm). *p < 0.05 represents statistically significant difference between control and treated groups in MTT assay. *p < 0.05 represents statistically significant difference between control and β-amyloid + ASH-WEX, β-amyloid + FIV, LPS + ASH-WEX, and LPS + FIV-treated groups. ^+^ *p* < 0.05 represents statistically significant difference between control and β-amyloid and LPS-treated group. ^#^ *p* < 0.05 represents statistically significant difference between β-amyloid and β-amyloid + ASH-WEX, β-amyloid + FIV, LPS and LPS + ASH-WEX, and LPS + FIV-treated groups

To determine the effective dose of ASH-WEX and FIV, BV-2 microglial cells were treated with different concentrations of ASH-WEX (0.1–2 %) and FIV (5–15 μg/ml) for 48 h and the cell viability was quantified by the conversion of yellow MTT into formazan crystals. The IC_50_ value for ASH-WEX and FIV was at 0.5 % and 15 μg/ml, respectively (Fig. 1A, i, ii; *p* ≤ 0.05). On the basis of this preliminary data, maximum nontoxic doses, i.e., 0.2 % and 10 μg/ml were used for ASH-WEX and FIV, respectively, for further experiments.

Both β-amyloid and LPS were used as the inflammatory triggers to activate the microglial cells. To determine their effective concentration, BV-2 microglial cells were treated with different concentrations of the β-amyloid (1–1000 nM) and LPS (0.01–10 μg/ml) for 48 h and immunostained for the cytoskeletal protein α-tubulin. On the basis of data obtained, 5 nM and 0.1 μg/ml were used as the effective noncytotoxic concentrations of β-amyloid and LPS, respectively, to activate the microglial cells.

### Effect of ASH-WEX and FIV on the morphology of the BV-2 microglial cells

Phase contrast images (Fig. 1B, row a, d) of both primary and BV-2 microglial cells showed significant change in morphology after 100 ng/ml LPS and 5 nM β-amyloid treatment. While treatment of BV-2 and primary microglial cells with either ASH-WEX or FIV did not induce any changes in their morphology, however, treatment of both ASH-WEX and FIV to LPS- and β-amyloid-exposed culture group inhibited activation of the microglia as was seen by normal morphology of these cells. Morphological changes in LPS- and β-amyloid-induced BV-2 cells were further confirmed by immunostaining for cytoskeletal protein α-tubulin, which further clearly showed that activated microglial cells had amoeboid morphology with thick processes and enlarged cell soma. ASH-WEX and FIV pretreatment prevented changes in morphology induced by LPS and β-amyloid in these cells which showed morphology similar to untreated cells (Fig. 1B, row b). We further confirmed the LPS- and β-amyloid-induced activation of the BV-2 and primary microglia at molecular level by studying expression of Iba-1 which is an ionized calcium-binding adaptor protein specifically expressed in the microglial cells in the brain. Among the different groups, LPS and β-amyloid treated both primary microglial cells and BV-2 microglial cells showed maximum expression of Iba-1 which was suppressed by pretreatment of these cells by ASH-WEX and FIV (Figs. 1B, row c; 3A, row a; 1a, iii; *p* ≤ 0.05) and more pronounced suppression was observed in FIV-treated group. Since ASH-WEX and FIV inhibited both LPS and β-amyloid, induced microglial-mediated neuroinflammation and activation seen in LPS-treated cultures were more prominent than the β-amyloid-treated cultures, so we further studied the expression of the various inflammatory mediators using LPS-stimulated primary microglial cells and BV-2 cell line.

### ASH-WEX and FIV inhibited the LPS-induced inflammatory pathway proteins

**Fig. 2:**
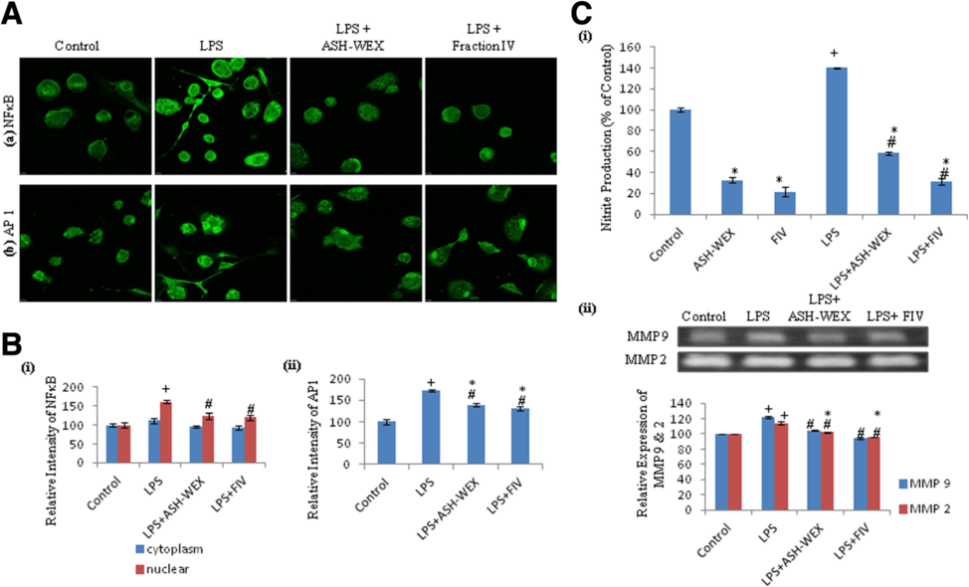
Inhibitory effects of ASH-WEX and FIV on LPS-induced inflammatory mediators’ expression in primary microglia. **A** Representative confocal images of activated primary microglial cells with or without pretreatment with ASH-WEX and FIV immunostained with (**a**) NFkB and (**b**) AP1. **B i**, **ii** Histograms represent the relative optical intensities of NFkB and AP1, respectively, in different treated groups as compared to control. **C i** Represents the relative production of the nitrite in different treated groups as compared to control due to neuroinflammation as estimated by the Griess reagent. **C ii** Representative MMP zymograms for control and different treated groups and histograms represent densitometric analysis of MMP bands. *p < 0.05 represents statistically significant difference between control and LPS + ASH-WEX and LPS + FIV-treated groups. ^+^ *p* < 0.05 represents statistically significant difference between control and LPS-treated group. ^#^ *p* < 0.05 represents statistically significant difference between LPS and LPS + ASH-WEX and LPS + FIV-treated groups

**Fig. 3:**
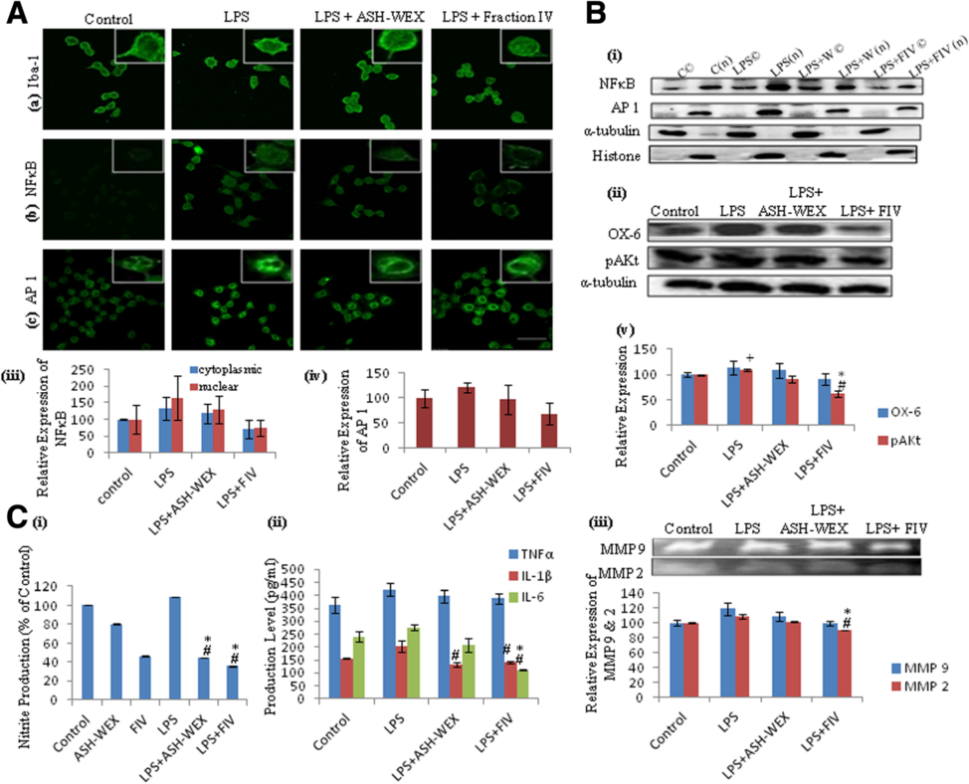
ASH-WEX and FIV pretreatment inhibits LPS-induced various inflammatory pathway protein expression in BV-2 microglia. **A** Representative confocal images of activated BV-2 microglial cells with or without pretreatment with ASH-WEX and FIV immunostained with (**a**) Iba-1, (**b**) NFkB, and (**c**) AP1. **B i** Representative Western blots of NFkB and AP1 showing inhibition of the translocation of the NFkB from cytoplasm to nucleus and decrease in expression of AP1 with ASH-WEX and FIV pretreatment. **B**, **iii**, **iv** Representative histograms present normalized relative expression of NFkB and AP1 plotted as mean ± SEM calculated from three independent experiments. Cytoplasmic expression of protein was normalized with α-tubulin, and nuclear expression was normalized with histone-3. **B ii** Representative Western blots of pAkt, OX-6 showing decrease in expression of pAkt, OX-6 with ASH-WEX and FIV pretreatment. **B v** Histogram representing mean of densitometric analysis of Western blot signals of pAkt, OX-6 after normalization with α-tubulin. **C** ASH-WEX and FIV inhibit the release of the inflammatory mediators due to neuroinflammation induced by LPS treatment. **C i** Represents the relative production of the nitrite in different treated groups as compared to control due to neuroinflammation as estimated by the Griess reagent. **C ii** Represents histograms depicting the decreased levels of the inflammatory cytokines (TNF-α, IL-1β, IL 6) by the ASH-WEX and FIV treatment as estimated by Sandwich ELISA. **C iii** Representative MMP zymograms for control and different treated groups and histograms represent densitometric analysis of MMP bands. *p < 0.05 represents statistically significant difference between control and LPS + ASH-WEX and LPS + FIV-treated groups. ^+^ *p* < 0.05 represents statistically significant difference between control and LPS-treated group. ^#^ *p* < 0.05 represents statistically significant difference between LPS and LPS + ASH-WEX and LPS + FIV-treated groups

As the change in the cellular morphology is associated with the functional activity of the microglial cells, we further studied the expression of various proteins associated with LPS-induced inflammatory pathways. Immunostaining for NFkB and AP1 showed enhanced expression of these proteins with higher nuclear translocation in LPS-treated primary microglial cells as compared to control. However, both ASH-WEX and FIV pretreatment to these cells downregulated the expression of these molecules and also their translocation to nucleus as depicted by the confocal images (Fig. 2A, row a and b) and their intensity plots (Fig. 2B, i, ii). Similarly, ASH-WEX and FIV also inhibited the expression of NFkB and AP1 in LPS-stimulated BV-2 microglial cells (Fig. 3A, row b and c). Immunostaining data was further confirmed by Western blot analysis of nuclear and cytoplasmic fractions of control and treated BV-2 microglial cultures which showed downregulation in nuclear expression of the NFkB and AP1 in ASH-WEX- and FIV-pretreated groups as compared to LPS alone (Fig. 3B, i, ii, iii). As microglial cells are key player in the immune response, we studied the expression of the major histocompatibility complex II (MHC II) as detected by OX-6 antibody. ASH-WEX- and FIV-pretreated BV-2 cells showed downregulated expression of the MHC II as compared to LPS alone treated cells (Fig. 3B, iv, v) indicating their role in the suppression of the microglial activation. PI3k-Akt pathway also plays an important role in inflammation and microglial activation. Western blot analysis showed that ASH-WEX and FIV pretreatment significantly downregulated the expression of pAkt^SER-473^ in these microglial cells as compared to LPS alone treatment group (*p* ≤ 0.05; Fig. 3B, iv, v).

### ASH-WEX and FIV inhibited the production of the various pro-inflammatory mediators

Activation of the microglial cells is generally accompanied by enhanced production of the iNOS which is responsible for further release of RNS. Thus, we further studied the RNS levels in control and ASH-WEX- and FIV-pretreated LPS groups using BV-2 and primary microglial cells. NO production was assayed by the Griess reagent. This reagent works on principle of azo coupling between diazonium species, which are produced from sulfanilamide in presence of NO_2_ and naphthylethylenediamine. ASH-WEX and FIV were found to inhibit the production of NO significantly in both primary (by 58 and 78 %, respectively) and BV-2 microglial cells (by 59 and 67 %, respectively), as compared to LPS-treated group (Figs. 2C, i and 3C, i; *p* ≤ 0.05). Activated microglia also produce various pro-inflammatory cytokines which ultimately lead to neuronal damage directly or indirectly. We detected amount of released inflammatory cytokines in conditioned media from all the treatment and control groups. ELISA test showed the highest production of inflammatory cytokines such as TNF-α, IL-1β, and IL-6 in LPS-treated BV-2 microglial cultures (17, 31, and 15 % more TNF-α, IL-1β, and IL-6, respectively, as compared to control). However, this LPS-induced production of inflammatory cytokines was significantly inhibited by ASH-WEX (up to 7 % TNF-α, 45 % IL-1β, 28 % IL-6) and FIV (up to 9 % TNF-α, 40 % IL-1β, 69 % IL-6) pretreatment as compared to LPS alone treated group (Fig. 3C, ii; *p* ≤ 0.05). These pro-inflammatory cytokines act as both primary as well as secondary mediators of neuroinflammation.

In the developing brain, during microglia-mediated inflammatory response, expression of the MMPs particularly gelatinases is concomitantly increased. To determine the production of MMPs, conditioned media from all the experimental groups were resolved in 10 % SDS-PAGE containing 0.1 % gelatine. ASH-WEX and FIV significantly inhibited LPS-induced release of the gelatinases MMP-9 and MMP-2 in both primary and BV-2 microglial cells as depicted by the gelatin zymogram data (Figs. 2C, ii and 3C, iii; *p* ≤ 0.05).

### ASH-WEX and FIV inhibited the release of the ROS

**Fig. 4:**
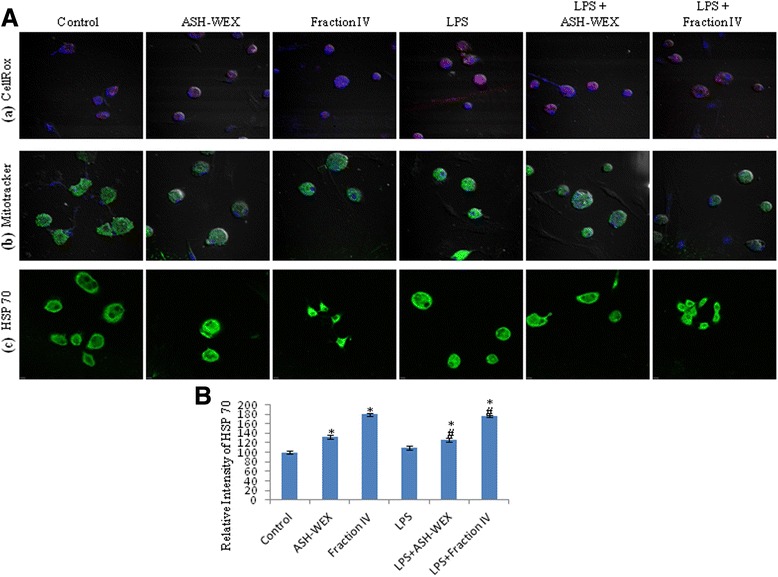
ASH-WEX- and FIV-mediated prevention of oxidative stress induced due to neuroinflammation in primary microglia. **A** Represents the confocal images of the (**a**) CellROX staining, (**b**) Mitotracker staining, and (**c**) HSP 70 immunostaining of the LPS-activated primary microglia with or without pretreatment with ASH-WEX and FIV illustrating the decreased levels of the reactive oxygen species and prevention of the mitochondrial turbulences induced due to the LPS treatment. **B** Histogram represents the relative optical intensity of HSP 70 in different treated groups as compared to control. *p < 0.05 represents statistically significant difference between control and LPS + ASH-WEX and LPS + FIV-treated groups. ^+^ *p* < 0.05 represents statistically significant difference between control and LPS-treated group. ^#^ *p* < 0.05 represents statistically significant difference between LPS and LPS + ASH-WEX and LPS + FIV-treated groups

**Fig. 5:**
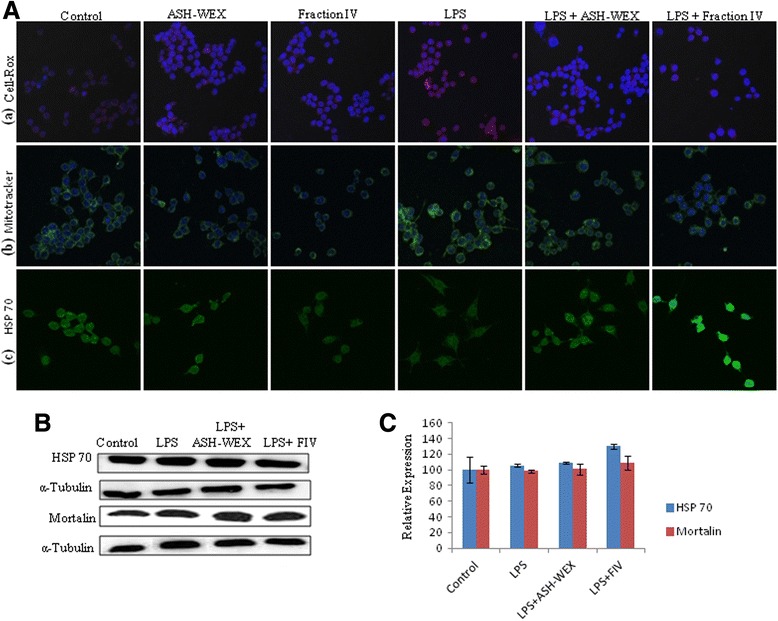
ASH-WEX and FIV prevent oxidative stress induced due to neuroinflammation in BV-2 microglial cells. **A** Represents the confocal images of the (**a**) CellROX staining, (**b**) Mitotracker staining, and (**c**) HSP 70 immunostaining of the LPS-activated BV-2 microglia with or without pretreatment with ASH-WEX and FIV illustrating the decreased levels of the reactive oxygen species and prevention of the mitochondrial turbulences induced due to the LPS treatment. **B** Representative Western blots of HSP 70, mortalin showing elevated levels of HSP 70, mortalin levels with ASH-WEX and FIV pretreatment. **C** Histogram presenting mean of densitometric analysis of Western blot signals of HSP 70, mortalin after normalization with α-tubulin

Since reactive oxygen and nitrogen species act as the critical signaling molecules to trigger the microglial-mediated inflammatory responses, thus, the levels of the ROS production were tested in all control and treatment groups using CellROX dye whose change in fluorescent intensity is the indicator of ROS production. ASH-WEX- and FIV-pretreated primary and BV-2 microglial cells showed lower red fluorescent intensity as compared to LPS alone treated cells which clearly indicates that pretreatment with ASH-WEX and FIV suppressed LPS-induced ROS production (Figs. 4A, row a; 5A, row a). As the main source of cellular ROS production is mitochondrial dysfunction, we further examined the effect of the microglial activation on mitochondrial activity using Mitotracker Green FM dye. The change in intensity of this dye indicates alteration in mitochondrial activity. LPS alone treated primary and BV-2 microglial cells showed higher mitochondrial stress as indicated by the highest fluorescence level in these cells. However, both ASH-WEX- and FIV-pretreated cultures showed reduced dye fluorescence indicating normal mitochondrial activity which may be the result of attenuated production of ROS (Figs. 4A, row b; 5A, row b). ASH-WEX and FIV pretreatment also upregulated the expression of stress chaperone HSP 70 in both primary (Fig. 4A, row c, B) and BV-2 microglia (Fig. 5A, row c, B, C), as detected by immunocytochemistry and Western blot data. However, we did not find any significant difference in expression of mitochondrial stress response protein mortalin in all the treatment groups in BV-2 microglial cells (Fig. 5B, C).

### ASH-WEX and FIV has the inhibitory effect on the BV-2 microglial cells migration

**Fig. 6:**
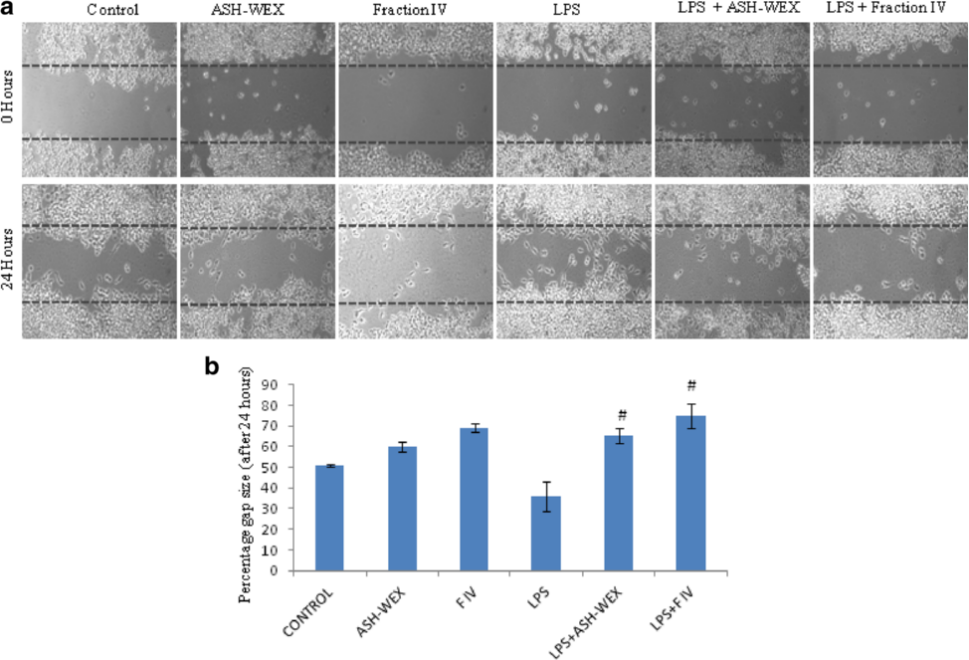
ASH-WEX and FIV attenuate the migration of the LPS-activated BV-2 microglia. BV-2 microglial cells were grown as a confluent monolayer, injured by applying a scratch and with ASH-WEX and FIV with and without treatment with LPS. **a** Representative phase contrast images which were taken at 0 and 24 h after scratching. **b** Histogram represents the percentage gap size after the 24 h (0 h gap was taken as 100 %). ^+^ *p* < 0.05 represents statistically significant difference between control and LPS-treated group. ^#^ *p* < 0.05 represents statistically significant difference between LPS and LPS + ASH-WEX and LPS + FIV-treated groups

Microglial cell migration is the hallmark of the inflammatory reactions, and chronic activation of the microglia is reported in various neurodegenerative diseases. Therefore, to determine the potential of the ASH-WEX and FIV to inhibit the microglial migration, scratch assay was performed using BV-2 microglial cells and migration into the cell-free scratch area was documented. Representative phase contrast images before and after treatment clearly showed that ASH-WEX and FIV pretreatment to the LPS-induced BV-2 microglial cells reduced their migration to the scratched area (Fig. 6a). The gap size was reduced to 36 % of the original gap size after 24 h (taking gap size at 0 h as 100 %) in LPS-treated BV-2 microglia. However, in ASH-WEX- and FIV-pretreated LPS-activated BV-2 microglia, gap size was 65 and 75 % of the original gap size after 24 h, respectively, indicating anti-migratory potential of the ASH-WEX and FIV (*p* ≤ 0.05; Fig. 6b).

### ASH-WEX and FIV caused the cell cycle arrest and apoptosis of the activated BV-2 microglial cells

**Fig. 7:**
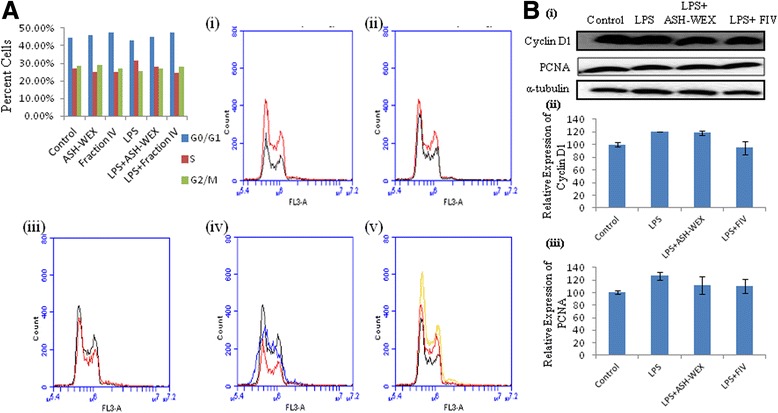
ASH-WEX and FIV suppresses proliferation of activated BV-2 microglial cells by causing cell cycle arrest. **A i**–**v** Represents plots showing the cell cycle analysis of the ASH-WEX- and FIV-treated BV-2 microglia with or without activation with LPS analyzed by PI stain using flow cytometer, and histogram presents the distribution of cells in G0/G1, S, and G2/M phases of cell cycle. **B i** Representative Western blot of Cyclin D1 and PCNA showing decrease in expression of Cyclin D1 and PCNA on ASH-WEX and FIV treatment. **B ii**, **iii** Histograms presenting mean of densitometric analysis of Western blot signals of Cyclin D1 and PCNA after normalization with α-tubulin. The data were obtained from three independent experiments

Activated microglial cells generally undergo proliferation; thus, we further studied whether ASH-WEX and FIV also arrest the cell cycle progression. The cell cycle distribution data revealed that treatment of the BV-2 microglia with LPS slightly increased their proliferation rate with 42.78 % cells in G_o_/G_1_, 31.63 % in S, and 25.59 % in G_2_/M phases of the cell cycle as compared to control cultures (44.55 % cells in G_o_/G_1_, 27.02 % in S, and 28.43 % in G_2_/M phases) (Fig. 7a, i–v). However, pretreatment of these cells with ASH-WEX inhibited this LPS-induced proliferation (G_o_/G_1_ (45.15 %), S (28.02 %), and G_2_/M (26.83 %) phases). This inhibition of proliferation was more pronounced in FIV-pretreated group (G_o_/G_1_ (47.39 %), S (24.51 %), and G_2_/M (28.10 %) phases). Consistent with these results, both extracts were also seen to inhibit the expression of Cyclin D1 which is required for the cell cycle progression (Fig. 7B, i, ii). The expression of another cell cycle regulatory protein PCNA, a DNA clamp loading protein, was also found to be downregulated in ASH-WEX- and FIV-pretreated groups (Fig. 7b, i, iii).

**Fig. 8:**
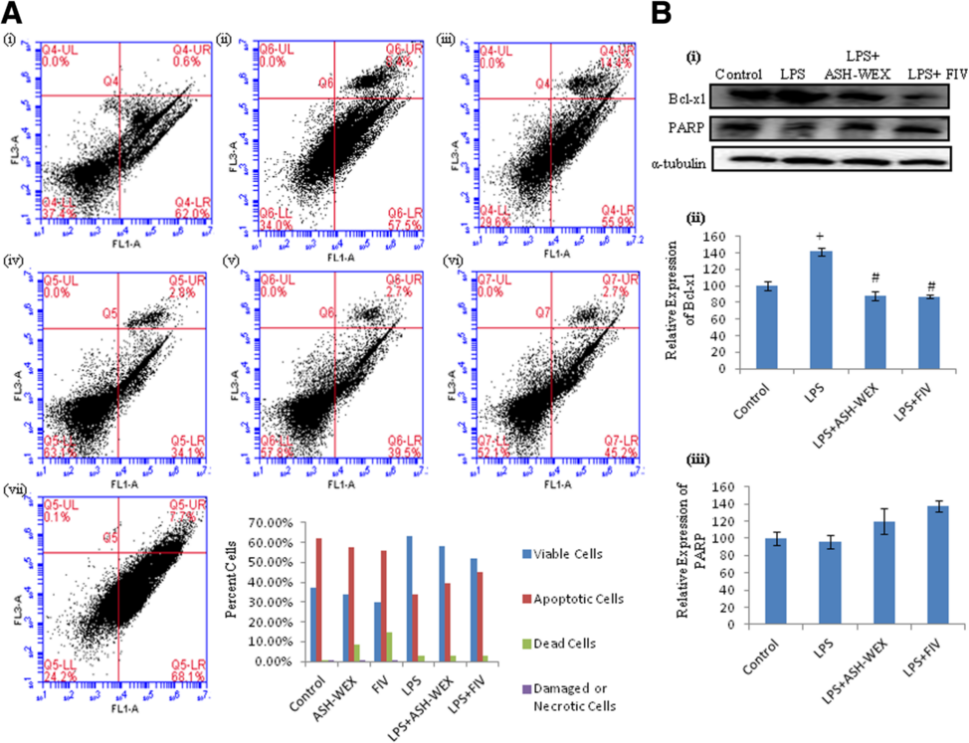
ASH-WEX and FIV induce the apoptosis of the LPS-activated BV-2 microglia. **A**, **i**–**vii** Represents the Annexin-FITC assay of the ASH-WEX- and FIV-treated BV-2 microglia with or without activation with LPS using flow cytometer. **B i** Representative Western blots of Bcl-xl and PARP showing decrease in expression of Bcl-xl and corresponding increase in the level of PARP release on ASH-WEX and FIV treatment. **B**, **ii**, **iii** Histograms presenting mean of densitometric analysis of Western blot signals of Bcl-xl and PARP after normalization with α-tubulin. The data were obtained from three independent experiments. ^+^ *p* < 0.05 represents statistically significant difference between control and LPS-treated group. ^#^ *p* < 0.05 represents statistically significant difference between LPS and LPS + ASH-WEX and LPS + FIV-treated groups

Further, to investigate whether ASH-WEX and FIV also induce apoptosis/pro-apoptosis of the activated BV-2 microglia, we performed the Annexin V-FITC assay. Treatment of the BV-2 microglia with LPS showed 63.07 % live cells and 34.06 and 2.85 % cells in the early and late apoptotic stage, respectively. Pretreatment of these cells with ASH-WEX and FIV showed more cells in apoptotic phase (39.48 % early apoptotic and 2.67 % late apoptotic in case of ASH-WEX) and (45.17 % early apoptotic and 2.72 % late apoptotic in FIV pretreatment group) and less number of the live cells (57.84 % in ASH-WEX and 52.12 % in FIV group) as compared to the LPS-treated microglia (Fig. 8a, i–v). Moreover, LPS treatment induced the expression of anti-apoptotic protein bcl-xl in BV-2 microglial cells, whereas ASH-WEX and FIV pretreatments were observed to significantly downregulate its expression (Fig. 8b, i, ii; *p* ≤ 0.05) and upregulation of expression of apoptotic cascade protein PARP (Fig. 8b, i, iii). This data suggest that pretreatment with ASH-WEX and FIV to LPS-activated BV-2 microglia may induce apoptosis of inflamed BV-2 microglial cells.

### Wit A and Withanone as the active components in ASH-WEX and FIV

**Fig. 9:**
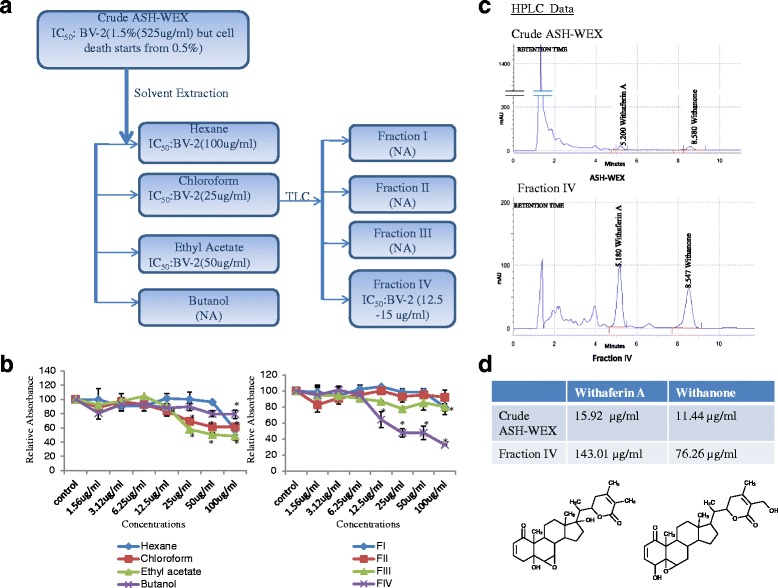
Bioactivity of the different fractions extracted from ASH-WEX and their characterization for the presence of active phytochemicals. **a** Flowchart representing the IC_50_ of the different fractions extracted from ASH-WEX. **b** Line graphs represent the cytotoxicity assay of the BV-2 microglia treated with different fractions after 48 h of the treatment. **c** HPLC chromatograms representing the presence of the bioactive components Withaferin A and Withanone in the ASH-WEX and FIV.*p < 0.05 represents statistically significant difference between control and treated groups. **d** Table representing the quantities of Withaferin A and Withanone in ASH-WEX and FIV and their biochemical structures

As ASH-WEX was seen to inhibit the microglial-mediated neuroinflammation, the fractionation and characterization of the ASH-WEX was performed to ascertain the bioactive components. ASH-WEX was subjected to a series of solvent extraction, and various soluble hexane, chloroform, ethylacetate, and butanol fractions were obtained (Fig. 9a). They were then assessed for cytotoxicity assay using BV-2 microglial cells. Out of the four fractions, chloroform fraction was found to be more active than the crude ASH-WEX. Later, chloroform fraction was further fractionated using thin-layer chromatography (mobile phase 97 hexane: 3 ethylacetate). Further, four fractions were obtained and evaluated for the cytotoxicity. Out of these, FIV was found to be the most active (Fig. 9b). Then, ASH-WEX and FIV were subjected to HPLC analysis for the possible bioactive molecules as compared to known standards from Ashwagandha. Both the extracts were found to contain Wit A and Withanone with more quantity and in suitable proportion (approx 2:1 ratio of Wit A and Withanone) in FIV.

## Discussion

Suppression of microglial-mediated neuroinflammation is considered as an important target in developing the therapeutics for the treatment of neurodegenerative diseases. The current study was aimed to study the anti-neuroinflammatory potential of the ASH-WEX and its active chloroform fraction (FIV). MTT assay revealed that treatment of the BV-2 microglial cells with ASH-WEX and FIV for 48 h reduced their proliferation rate, since ASH-WEX and FIV treatment decreased the cell viability beyond the concentration ≥0.5 % and ≥15 μg/ml, respectively, in dose dependent manner. So 0.2 % of ASH-WEX and 10 μg/ml of FIV were chosen as the effective dose for further experiments. BV-2 and primary microglia activated with both 100 ng/ml of bacterial endotoxin LPS and 5 nm of β-amyloid were used as in vitro model system for preliminary experiments.

In the adult brain, microglia exhibit ramified morphology and are involved in immune surveillance. In response to the immunological insult, these quiescent microglial cells retract their ramifications, transforming into an amoeboid-like cells with motile protrusions responsible for its inflammatory phenotype [27]. α-Tubulin immunostaining and phase contrast images showed that ASH-WEX and FIV inhibited both LPS- and β-amyloid-induced amoeboid morphology of these cells and restored their ramified morphology. At the molecular level, ASH-WEX and FIV pretreatment significantly suppressed the expression of the microglial-specific marker Iba-1 which was upregulated in both LPS- and β-amyloid-treated BV-2 and primary microglial cells. Iba-1, 17 kDa calcium-binding adaptor protein, has been reported to be upregulated during inflammation which helps to discriminate between the surveilling and activated microglia [28, 29]. These observations provided the first line of evidence that ASH-WEX and FIV inhibited LPS- and β-amyloid-induced microglial activation xand suppressed neuroinflammation. Further, ASH-WEX- and FIV-pretreated BV-2 microglial cells also showed the downregulated expression of the MHC II compared to LPS-treated BV-2 microglial cells as detected by OX-6 immunostaining.

LPS-induced activation of both primary and BV-2 microglial cells was seen to increase the production of ROS (as detected by CellROX staining) which was associated with higher mitochondrial activity as evident from increased fluorescence of Green FM dye. The mitochondrial dysfunction plays important role in the ROS production in various models of chronic inflammation [30, 31]. LPS-treated primary and BV-2 microglial cells also showed higher level of nitrite released in the media which is already reported to cause upregulation of iNOS and results in the synthesis of high levels of NO [32]. Interestingly, pretreatment with ASH-WEX and FIV suppressed the effects of LPS as both ASH-WEX and FIV were observed to inhibit production of ROS and nitrite, thus resulting in reduced mitochondrial stress. It may be suggested that both extracts may prevent the oxidative damage during neuroinflammation by regulating the mitochondrial homeostasis. Various previous studies also reported anti-oxidative potential of *Withania* leaf extract in physiological abnormalities seen in the PD [33]. These observations are also supported by the upregulated expression of the mitochondrial stress response proteins like HSP 70 and mortalin in the ASH-WEX- and FIV-pretreated LPS-activated microglia. The overexpression of these chaperone proteins are known to protect the mitochondria and reduce oxidative stress [34, 35]. Upregulation of HSP 70 is also reported to suppress LPS-induced cytokine expressions by inhibiting the IkBα degradation and NFkB nuclear translocation [36]. Shikonin is reported to attenuate the production of NO/iNOS, TNF-α, IL-1β, and COX-2 in LPS-stimulated microglia by inhibiting Akt phosphorylation [37].

Enhanced production of ROS amplifies the inflammatory reaction and contributes to the subsequent neuronal damage in neurodegenerative diseases. This was evident from the sandwich ELISA-based immunoassay data showing dramatic increase in TNF-α, IL-1β, and IL-6 production in LPS-treated microglial cells. This increase in the release of these cytokines was significantly inhibited by ASH-WEX and FIV pretreatment to these LPS-treated cells. Experiments carried out in vitro and in vivo have demonstrated that TNF-α promotes neurodegeneration of dopaminergic neurons in the substantia nigra pars compacta (SNpc) in PD [38]. Experiments using different mutant mice deficient in iNOS or TNFα receptors have shown reduced neurotoxicity [39, 40]. A recent study reported that LPS activates the microglia with immediate superoxide release and later enhances the production of TNF-α, NO, prostaglandin E_2_ (PGE_2_), and IL-1β [41]. The current data may suggest that ASH-WEX and FIV inhibited microglial-mediated neuroinflammation by preventing the release of the ROS, RNS, and pro-inflammatory cytokines which work synergistically to induce the neurotoxicity and increase the duration of the chronic inflammation [42]. Wit A also has been reported to inhibit the iNOS expression and NO production in Raw 264.7 cells [43].

LPS-induced inflammatory cascades in microglial cells include activation of pathways such as NFkB, MAPK, and PI3K-Akt pathways, which have high implication in neurodegenerative processes [23]. Downregulated expression of the transcription factor NFkB in the ASH-WEX- and FIV-pretreated cells was also accompanied by inhibition of its translocation from cytoplasm to nuclear compartment. NFkB activation and its translocation are critical for the expression of the various cytokines, chemokines, receptors required for neutrophil adhesion and migration, MHCs, iNOS, and COX-2 in microglia in response to LPS [44, 45]. Our results are in line with the recent reports suggesting that anti-inflammatory activity of 30 % methanolic extract of the *Withania* leaves in stainless steel implant induced inflammation in zebrafish by inhibiting the NFkB transcriptional activity [46]. Further, its active component, i.e., Wit A has also been reported to inhibit the NFkB activity in cellular model of cystic fibrosis inflammation [47].

ASH-WEX and FIV pretreatment to these microglial cells also inhibited LPS-induced AP1 expression. The transcriptional activation of the AP1 via JNK/MAPK pathway has been reported to induce the production of the pro-inflammatory factors in LPS-activated microglia [48, 49]. The concerted action of both AP1 and NFkB signaling pathways together results in the induction of pro-inflammatory cytokines [50, 51]. Therefore, simultaneous inhibition of both NFkB and AP1 transcriptional activity by ASH-WEX and FIV may be responsible for their anti-neuroinflammatory potential of ASH-WEX and FIV. Many other plant products like brevicompanine, curcumin, and isooreintin have been reported to attenuate the LPS-induced production of the inflammatory mediators through inhibiting both NFkB and AP1 pathways [52–54]. Further, ASH-WEX- and FIV-pretreated cells also showed significant downregulation in the expression of p-Akt^SER473^ as compared to LPS alone treated group. Phosphorylation of Akt leads to changes in the catalytic activity of downstream targets, such as GSK-3, mTOR, COX-2, and mPGES-1, and results in secretion of various prostaglandins and leukotrienes having inflammatory activity [55–57].

During any insult to the brain or injury, the activation of microglia leads to induction of proliferation and migration of these cells towards the site of the lesion with elevated production of the various inflammatory molecules [58, 59]. ASH-WEX and FIV pretreatment was observed to downregulate the expression of the cell cycle regulatory proteins PCNA and Cyclin D1 along with cell cycle arrest of the LPS-activated BV-2 microglia at G_0_/G_1_ and G_2_/M phase. The inhibition of proliferation was also accompanied by induction of apoptosis as depicted by AnnexinV-FITC flow cytometry data. ASH-WEX- and FIV-mediated induction of apoptosis may be the result of inhibition of anti-apoptotic protein Bcl-xl. Upregulated Bcl-xl expression has been detected in reactive microglia of the patient with neurodegenerative diseases [60]. Both the extracts were also seen to elevate the levels of the PARP expression, whose cleavage into 27 and 87 kDa fragments leads to the apoptosis progression. Apoptosis of the inflamed microglial cells is one of the mechanisms of the anti-inflammatory compounds to inhibit the neuroinflammation [61].

Further, ASH-WEX and FIV also inhibited the production of the MMP 2 and MMP 9 suggesting their role in maintaining the blood-brain barrier (BBB) integrity as illustrated by gelatin zymogram data. MMPs are microglial inflammatory factors that degrade components of the basal lamina, leading to the disruption of the BBB, and contribute to the neuroinflammatory response in many neurological diseases [62]. Further released proteases from activated microglia result in proteolytic degradation of the BBB which further enhances the recruitment of the microglia to the lesion site [63], as depicted by maximum migration of cells to the scratched area in LPS-treated cultures. ASH-WEX and FIV were observed to reduce rate of migration of cells in scratched area which may also be due to collective effect of the controlled proliferation, apoptosis, and cell cycle arrest by the ASH-WEX and FIV pretreatment. Based on these observations, it may be suggested that ASH-WEX and especially FIV may prove to be potential agent for preventing the uncontrolled migration of the microglia to the lesion site during CNS injury.

## Conclusions

**Fig. 10:**
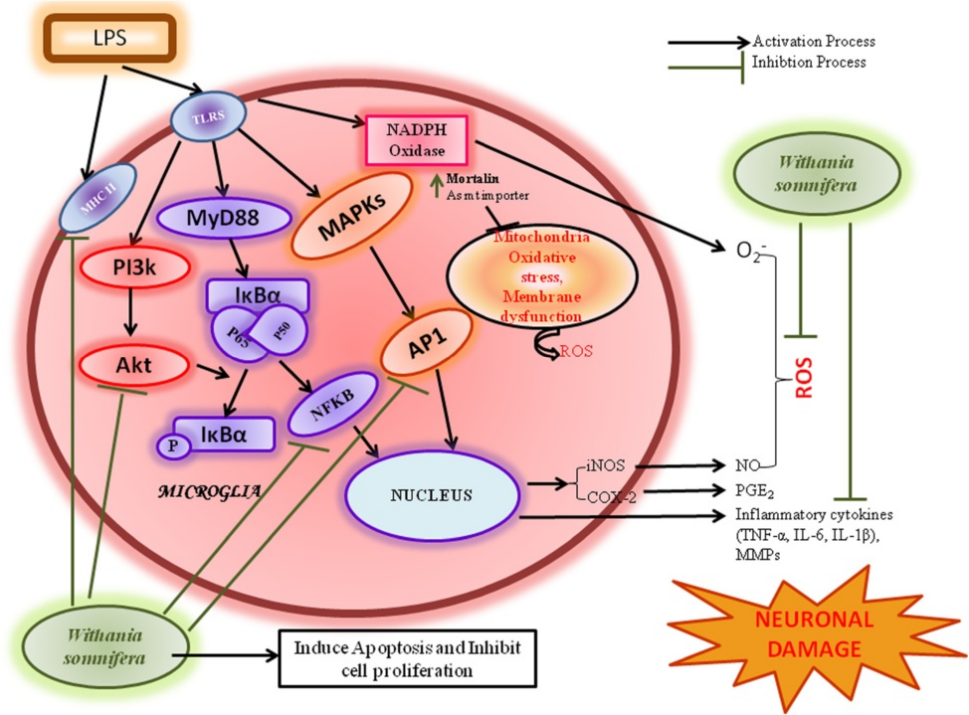
Graphical representation of the possible inflammatory cascades or proteins involved in the anti-neuroinflammatory potential of the ASH-WEX and FIV

In the light of the present study, it may be concluded that ASH-WEX and one of its active chloroform fraction (FIV) prevents the microglial-mediated neuroinflammation by inhibiting the production of various pro-inflammatory mediators via various inflammatory pathways (Fig. 10). They also control the microglia proliferation and migration and led cells to undergo apoptosis. Furthermore, FIV was more potent anti-inflammatory agent than the crude ASH-WEX. We have also characterized the ASH-WEX and its active chloroform fraction (FIV) for the presence of bioactive components using the reverse phase HPLC method. Interestingly, both extracts were found to contain Wit A and Withanone, but FIV seems to contain more quantity of Wit A and Withanone in suitable proportion than ASH-WEX (Fig. 9). This may explain the higher effectiveness of the FIV than the ASH-WEX in the current results. The anti-neuroinflammatory activity of the ASH-WEX and FIV might be due to the combination of Withanone and Wit A as active components. In view of the current findings, the extract from this plant may prove to provide safe and effective alternative to the conventional NSAIDs used for the treatment of the neurodegenerative diseases.

## Abbreviations

AP1, activator protein 1; ASH-WEX, Ashwagandha leaf water extract; DIC, differential interference contrast; FITC, fluorescein isothiocyanate; FIV, fraction IV; LPS, lipopolysaccharide; IL-1β, interleukin-1 β; MMPs, matrix metalloproteinases; MHC II, major histocompatibility complex II; MTT, 3-(4,5-dimethylthiazol-2-yl)-2,5-diphenyl tetrazoliumbromide; NFkB, nuclear factor-kB; PCNA, proliferating cell nuclear antigen; PBS, phosphate-buffered saline; RNS, reactive nitrogen species; ROS, reactive oxygen species; SDS, sodium dodecyl sulfate; SEM, standard error of the mean; TNF-α, tumor necrosis factor-α
